# Supplementary material for: Patient reported outcome measures relevant to asthma remission: scoping review protocol
Source: MethodsX. 2025 Oct 15;15:103679. doi: 10.1016/j.mex.2025.103679 (PMC12590250; doi:10.1016/j.mex.2025.103679)
Supplement: Supplementary file 1 — Supplementary material and/or additional information [OPTIONAL] [file mmc1.docx]

**Article information**

**Article title**

Patient Reported Outcome Measures Relevant To Asthma Remission: Scoping Review Protocol

**Authors**

Allison Michaud*^1,2^, John Politis^1^, Lachlan Faktor^1^, Philip G Bardin^1^, Amy HY Chan^3^^, Paul Leong (0000-0002-7409-9328)^1^^

**^co-senior author**

**Affiliations**

^1^Monash Lung Sleep Allergy and Immunology and School of Clinical Sciences, Monash Health and Hudson Institute of Medical Research, Monash University, Melbourne, VIC, Australia; ^2^Division of Respirology, Department of Medicine, University of Calgary, Calgary, Canada; ^3^School of Pharmacy, Faculty of Medical and Health Sciences, University of Auckland, Auckland, New Zealand.

**Corresponding author’s email address and Twitter handle**

Dr Allison Michaud PhD MD

Monash Lung Sleep Allergy and Immunology, Monash University and Medical Centre, 246 Clayton Road, Clayton, Victoria, 3168, Australia and

Division of Respirology, Department of Medicine

University of Calgary, 2500 University Drive NW, Calgary, Canada, T2N 1N4

Telephone: +61 (3) 9594 2900 and +1 (403) 943-8658

Fascimile:+61 (3) 9594 6032 and +1 (403) 943-8666
Email: Allison.michaud@ahs.ca

**Keywords**

***Disease remission assessment,*** ***Asthma patient experience****,* ***Chronic illness measurement tools***

*.*

**Related research article**

*None*

***For a published article:***

**None**

**Abstract**

**Introduction**
Asthma affects over 260 million people globally. Recent advances in asthma care have highlighted remission as a key treatment goal. While remission requires agreement between patients and healthcare providers, there is no standard way to assess the patient experience of remission. This scoping review aims to identify validated Patient Reported Outcome Measures (PROMs) that capture patient experiences of disease remission and may be applicable to asthma. Determining items and domains that are most important to patients will inform the development of a conceptual framework for a PROM for asthma remission.

**Methods and Analysis**

- This review will identify PROMs that quantify remission in long-term diseases.
- It will follow the Joanna Briggs Institute Manual and the PRISMA-ScR guidelines.
- Two independent reviewers will screen titles and abstracts following a training and calibration phase.
- Data extraction will also be performed independently by two authors, with disagreements resolved through discussion or a third reviewer.

**Ethics and Dissemination**
No ethics approval is required as no human participants are involved. Findings will be shared at academic conferences and published in peer-reviewed journals.

**Graphical abstract**

*Separately included*

**Specifications table**

| **Subject area** | Medicine and Dentistry |
| --- | --- |
| **More specific subject area** | Asthma |
| **Name of your protocol** | Patient Reported Outcome Measures Relevant To Asthma Remission: Scoping Review Protocol |
| **Reagents/tools** | Not applicable |
| **Experimental design** | Not applicable |
| **Trial registration** | Open Science Framework (registry number osf.io/fyz8j) |
| **Ethics** | Not applicable |
| **Value of the Protocol** | 1. Current definitions of asthma remission lack the patient perspective of remission and there is no definitive way to assess patient reported remission currently 2. Current asthma questionnaires are framed on disease control rather than remission 3. This protocol will identify themes used in other diseases which can be used to develop a patient reported outcome measure for asthma |

**Background**

Asthma is a long-term disease of international importance that affected over 260 million people globally and caused 455 000 deaths in 2019.^1^ Recent therapeutic advances mean that unprecedented and transformational improvements in patient outcomes are now possible. This new era of asthma management has highlighted the notion that the concept of asthma control requires extension to define and encompass remission. Taken together with advances in knowledge on lung function trajectories,^2^ this progress implies that novel asthma treatment paradigms aimed at achieving remission may improve lifetime outcomes for people with asthma. It is not clear, however, how remission would be measured and operationalised.

Remission is a new concept in the field of asthma.^3^ While children can commonly “grow out” of mild asthma without need for chronic inhalers (spontaneous remission), remission of severe asthma was more rare prior to the development of advanced therapies such as biologics. A number of definitions exist for clinical asthma remission,^4^ which is continually evolving but typically includes at minimum an absence of asthma symptoms and exacerbations. Biologic remission additionally includes low type 2 inflammatory biomarkers, including resolution of blood eosinophilia, normalization of exhaled nitric oxide fraction (FeNO), and normal airway responsiveness.

The most well-established consensus document^5^ on clinical remission established four criteria: sustained absence of substantial asthma symptoms, stabilized lung function, no systemic corticosteroid requirements and, critically, patient and healthcare provider agreement regarding remission. Regardless of the definition used for remission, the patient experience of remission is a crucial part of the definition. However, the final aspect of this definition is problematic as there is no way to currently assess remission from the patient perspective. Asthma questionnaires have been historically created to measure disease control^6-9^, rather than remission, and as such they typically focus on respiratory symptoms and limitation as well as reliever use. It is possible these will have a ceiling effect where remission is concerned, or may not examine domains that are specifically important for asthma remission.

Patient reported outcomes measures (PROMs) are “a measurement of any aspect of a patient’s health status that comes directly from the patient (i.e., without the interpretation of the patient’s responses by a physician or anyone else)”.^10^ How these relate to remission in asthma is currently unclear. While some fields of medicine such as oncology have patient-agnostic remission definitions based on molecular or imaging, these test-based criteria are unlikely to be sufficient for asthma, where test results and patient experience can be discordant.^11^ In other long-term inflammatory conditions such as rheumatoid arthritis, research indicates that the concept of clinical ‘remission’ tends to focus on symptoms rather than on inflammatory pathologies and that patients and clinicians have different ideas of what constitutes remission.^12, 13^ It is therefore important to establish using structured measures what clinical remission means to patients using patient-reported measures.

Research priorities for patient reported outcome measure in asthma remission have recently been described^14^ and a cornerstone is to identify a process or framework by which patients and health-care providers may agree on remission. The aim of this scoping review is to identify validated PROMs that capture patient experiences of the concept of disease remission in long-term conditions, which may inform the development of a future PROM to measure the patient experience of asthma remission. As there are currently no PROMs that have been validated in asthma remission, the review will synthesise literature that reports on PROMs that quantify the patient experience of remission in any long-term condition.

**Description of protocol**

**METHODS AND ANALYSIS**

This scoping review began in October 2024 and is anticipated to finish in October 2025. The protocol was finalized and submitted for publication prior to Title and Abstract screening. The proposed scoping review will be conducted in accordance with the JBI methodology for scoping reviews^15^ and reported in line with the Preferred Reporting Items for Systematic Reviews and Meta-Analyses extension for Scoping Reviews (PRISMA-ScR).^16^ This protocol has been registered on Open Science Framework (registry number osf.io/fyz8j) as recommended by PRISMA-ScR.

**Patient And Public Involvement**

Patients and the general public have not been involved in the design of the scoping review. The results of the review will be presented to an Asthma Consumer Advisory Group based in Australia that is comprised of diverse members with different asthma severities and experiences. The group will be able to provide feedback on identified themes and their relation to the patient experience of asthma remission. Patients will be involved in the future design and development of a PROM for remission in asthma .

**Eligibility Criteria:**

This review will include studies that involve patients of any age with a long term condition for which PROMs were used to capture the concept of remission from the patient perspective. Given that asthma can affect patients at any age, no age limitations were included. This scoping review will consider quantitative, qualitative, and mixed methods study designs for inclusion to to comprehensively map out the breadth and depth of existing research. Only primary studies published in indexed journals will be considered. Review articles, editorials, and letters will be excluded as they are unlikely to describe the development of individual PROMs. Conference abstracts will also be excluded as data are unlikely to be reported in sufficient detail to inform the development of a remission PROM. Grey literature will be excluded due to the broad nature of the topic and anticipated variable quality of PROM assessment in this body of literature.

**Search Strategy:**

Keywords and index terms were identified from previous literature and with the help of a health sciences librarian and used to conduct a search in Ovid MEDLINE (Table 1). The search terms were then adapted to develop a full search strategy for Ovid Embase, Ovid PsycINFO, Clarivate Web of Science, and EBSCO CINAHL Plus to identify additional articles related to the research topic. No restriction on language or year of publication was applied. The reference list of articles for full text review will be screened for additional papers with forward and backward chasing using established software. The initial search for this study was conducted in October 2024.

**Table 1**. Search terms for Ovid MEDLINE, to be mirrored in other databases.

| Number | **Strategy/Medical Subject Headings (MeSH)** |
| --- | --- |
| 1 | exp Remission Induction/ |
| 2 | exp Remission, Spontaneous/ |
| 3 | disease free.mp. |
| 4 | remission.mp. |
| 5 | disease-free survival/ |
| 6 | 1 or 2 or 3 or 4 or 5 |
| 7 | exp Patient Reported Outcome Measures/ |
| 8 | patient reported outcome*.mp. |
| 9 | patient centered outcome*.mp. |
| 10 | patient centred outcome*.mp. |
| 11 | patient perspective.mp. |
| 12 | patient perception.mp. |
| 13 | (perception adj3 remission).mp. |
| 14 | (patient reported adj3 remission).mp. |
| 15 | patient defined remission.mp. |
| 16 | 7 or 8 or 9 or 10 or 11 or 12 or 13 or 14 or 15 |
| 17 | 6 and 16 |

**Study Selection:**

Following the search, all identified records will be collated and uploaded into Covidence software (Covidence, Melbourne, Australia) and duplicates removed. Titles and abstracts will then be screened by two independent reviewers for assessment against the inclusion criteria. Reviewer training and calibration will precede the selection process which includes the initial assessment of at least 15 randomly chosen titles and abstracts in the first stage followed by at least 10 full-text articles, until an acceptable level of inter-rater reliability is reached based on consensus discussion. The full text of selected citations will be assessed in detail against the inclusion criteria by two independent reviewers. Any disagreements that arise between the reviewers at each stage of the selection process will be resolved through discussion or with a third reviewer if necessary. The results of the search will be reported in full in the final scoping review and presented in a PRISMA flow diagram.^17^

**Data Extraction:**

Data will be extracted from papers included in the scoping review by 2 independent reviewers using Covidence. The data extracted will include specific details about the study population, country, setting, underlying disease process, definition of remission, PROM name and version, year developed, language, mode and method of administration, number of items and domains, outcomes, feasibility, patient acceptability, interpretability, and psychometric properties. Any disagreements that arise between the reviewers will be resolved through discussion or with a third reviewer, as required. Authors of papers will be contacted by email up to a maximum of two times, at least two weeks apart, to request missing or additional data, where required.

**Data Analysis and Presentation:**

Identified evidence will be extracted and a qualitative synthesis developed in Word. We will summarize relevant data with the use of tables and graphs. A narrative synthesis will be performed to highlight pertinent PROMs in remission and how components may be applied to the topic of remission in asthma.

**Ethics And Dissemination:**

As this review does not involve human participants, no ethics approval is required. The results of this review will be presented at academic conferences and published in peer-reviewed journals. This review is part of a broader project that aims to identify variables that can be used as PROMs in asthma remission.

**Protocol validation**

*Not applicable*

**Limitations**

*Not applicable*

**CRediT author statement**

All authors fulfil ICMJE criteria for authorship. AM, PGB, AHYC, and PL contributed to initial conceptualization and methodology, with JP and LF contributing to design revisions. AM is the guarantor of the study and is responsible for data curation, project administration and leading formal analysis. PL and AM wrote the first draft of the protocol. All authors critically reviewed its content, approved the final protocol manuscript for publication and agree to be accountable for the work. No artificial intelligence assistance was used at any stage.

**Acknowledgments**

This research did not receive any specific grant from funding agencies in the public, commercial, or not-for-profit sectors.

**Declaration of interests**

*Please* ***tick*** *the appropriate statement below (please do not delete either statement) and declare any financial interests/personal relationships which may affect your work in the box below.*

The authors declare that they have no known competing financial interests or personal relationships that could have appeared to influence the work reported in this paper.

The authors declare the following financial interests/personal relationships which may be considered as potential competing interests:

AM has received honoraria from Valeo Pharma.

JP and LF have no disclosures of interest.

PB has served on Advisory Boards and provided educational lectures for GlaxoSmithKline, AstraZeneca, Sanofi and Chiesi. Honoraria are donated to a charitable research institute.

AHYC reports research grants from Health Research Council of New Zealand, Asthma UK, University of Auckland, Oakley Mental Health Foundation, Chorus Ltd, World Health Organisation, and Hong Kong University, outside the submitted work and all paid to her institution (the University of Auckland). AC previously held the Robert Irwin Postdoctoral Fellowship and is the current recipient of the Auckland Medical Research Foundation Senior Research Fellowship. AHYC also reports consultancy fees from AcademyeX and Spoonful of Sugar Ltd, travel support from AstraZeneca, and was previously on the Board of Asthma NZ. She is a member of Respiratory Effectiveness Group (REG), member of the Scientific Advisory Board for Asthma Respiratory Foundation NZ, international member of the Pharmacy Respiratory Task Force, and working group lead for the European Respiratory Society Clinical Research Collaboration “CONNECT”.

PL has received honoraria from GlaxoSmithKline, AstraZeneca and Chiesi.

**Supplementary material *and/or* additional information [OPTIONAL]**

Nil

**References**

1. Vos T, Lim SS, Abbafati C, et al. Global burden of 369 diseases and injuries in 204 countries and territories, 1990-2019: a systematic analysis for the Global Burden of Disease Study 2019. The Lancet. 2020;396(10258):1204-22.

2. Okyere DO, Bui DS, Washko GR, et al. Predictors of lung function trajectories in population-based studies: A systematic review. Respirology. 2021;26(10):938-59.

3. Upham JW, James AL. Remission of asthma: The next therapeutic frontier? Pharmacol Ther. 2011;130(1):38-45.

4. Lommatzsch M. Remission in asthma. Curr Opin Pulm Med. 2024;30(3):325-9.

5. Menzies-Gow A, Bafadhel M, Busse WW, et al. An expert consensus framework for asthma remission as a treatment goal. Journal of Allergy and Clinical Immunology. 2020;145(3):757-65.

6. Nathan RA, Sorkness CA, Kosinski M, et al. Development of the asthma control test: a survey for assessing asthma control. Journal of Allergy and Clinical Immunology. 2004;113(1):59-65.

7. Juniper EF, Bousquet J, Abetz L, et al. Identifying ‘well-controlled’and ‘not well-controlled’asthma using the Asthma Control Questionnaire. Respiratory medicine. 2006;100(4):616-21.

8. Juniper EF, O'Byrne PM, Guyatt GH, et al. Development and validation of a questionnaire to measure asthma control. Eur Respir J. 1999;14(4):902-7.

9. Murphy KR, Chipps B, Beuther DA, et al. Development of the Asthma Impairment and Risk Questionnaire (AIRQ): A Composite Control Measure. The Journal of Allergy and Clinical Immunology: In Practice. 2020;8(7):2263-74.e5.

10. Acquadro C, Berzon R, Dubois D, et al. Incorporating the patient's perspective into drug development and communication: an ad hoc task force report of the Patient-Reported Outcomes (PRO) Harmonization Group meeting at the Food and Drug Administration, February 16, 2001. Value Health. 2003;6(5):522-31.

11. Schifano ED, Hollenbach JP, Cloutier MM. Mismatch between asthma symptoms and spirometry: implications for managing asthma in children. J Pediatr. 2014;165(5):997-1002.

12. Trivedi I, Darguzas E, Balbale SN, et al. Patient Understanding of "Flare" and "Remission" of Inflammatory Bowel Disease. Gastroenterol Nurs. 2019;42(4):375-85.

13. van Tuyl LHD, Boers M. Rheumatoid arthritis: Remission - keeping the patient experience front and centre. Nat Rev Rheumatol. 2017;13(10):573-4.

14. Chan AHY, Leong P, Politis J, et al. Patient-reported outcomes: missing in asthma remission. Lancet Respir Med. 2024;12(9):665-6.

15. Peters M, Godfrey C, McInerney P, et al. Scoping reviews. 2024. In: Aromataris E, Lockwood C, Porritt K, Pilla B, Jordan Z, editors JBI Manual for Evidence Synthesis JBI; 2024 [cited 2024 10 18] Available from: <https://synthesismanualjbiglobal> <https://doiorg/1046658/JBIMES-24-01> [Internet].

16. Tricco AC, Lillie E, Zarin W, et al. PRISMA Extension for Scoping Reviews (PRISMA-ScR): Checklist and Explanation. Ann Intern Med. 2018;169(7):467-73.

17. Page MJ, McKenzie JE, Bossuyt PM, et al. The PRISMA 2020 statement: an updated guideline for reporting systematic reviews. BMJ. 2021;372:n71.
